# Fluoride Levels in Austrian Drinking Water Are Insufficient for Effective Caries Prevention

**DOI:** 10.3390/dj13100446

**Published:** 2025-09-29

**Authors:** Alice Blufstein, Elias Salzmann, Bledar Lilaj, Rinet Dauti, Oleh Andrukhov, Andrea Nell

**Affiliations:** 1 Competence Center for Periodontal Research, University Clinic of Dentistry, Medical University of Vienna, 1090 Vienna, Austria; oleh.andrukhov@meduniwien.ac.at; 2 Clinical Division of Periodontology, University Clinic of Dentistry, Medical University of Vienna, 1090 Vienna, Austria; elias.salzmann@meduniwien.ac.at; 3 Clinical Division of Conservative Dentistry, University Clinic of Dentistry, Medical University of Vienna, 1090 Vienna, Austria; bledi.lilaj@gmail.com (B.L.); rinet.dauti@gmail.com (R.D.); nell.andrea@icloud.com (A.N.)

**Keywords:** fluorides, drinking water, dental caries, preventive dentistry, Austria

## Abstract

**Background/Objectives**: Fluorides play a well-established role in preventing dental caries, primarily by enhancing enamel resistance and inhibiting demineralization. Drinking water is among the most important sources of systemic fluoride intake. In 1993 and 2007, national analyses of Austrian drinking water revealed fluoride levels below 0.5 mg/L in almost all regions, which is insufficient for effective caries prevention. The present study aimed to re-examine the fluoride concentration in Austrian drinking water. **Methods**: Drinking water was collected in a total of 1985 Austrian municipalities. Fluoride concentration was measured by a fluoride-selective electrode. **Results**: The average fluoride concentration in the measured water samples ranged between 0.1 and 0.27 mg/L, depending on the region. The analysis revealed that 98% of the municipal drinking water samples contained fluoride at concentrations below 0.5 mg/L. In almost one quarter of Austrian municipalities, the fluoride levels amounted to less than 0.1 mg/L. The fluoride concentration in the drinking water of one Tyrolean municipality exceeded the recommended threshold. **Conclusions**: The results of the study reveal that the fluoride concentration in Austrian drinking water is generally too low to provide effective prevention against dental caries, affecting nearly all municipalities. Notably, the drinking water of one municipality reached potentially harmful fluoride levels. These findings could be used as a basis for targeted and individual fluoride supplementation, as well as for national or area-specific guidelines.

## 1. Introduction

Although dental caries is considered a largely preventable and controllable disease, its prevalence is still increasing globally and remains a major public health challenge [1]. In 2024, caries prevalence in 6- to 7-year-old children in Austria was 42%, which remains far from the World Health Organization (WHO) target of 80% caries-free children, corresponding to a maximum prevalence of 20% [2,3]. While there is no recent data for Austrian adults, the WHO reports a caries prevalence of 33.6% among the adult population in Europe in 2023. Apart from oral hygiene measures, the use of fluorides constitutes one of the most effective approaches to preventing dental caries [4]. Not only does it promote remineralization and prevent demineralization of teeth, but it also effectively inhibits the activity of cariogenic bacteria in dental plaque [5]. Fluorides can be applied either topically in the form of toothpastes, mouthwashes, varnishes, and gels, or systemically by the intake of tablets, lozenges, salts, and drinking water [6].

The fluoride concentration in drinking water depends on various factors, such as pH, temperature, the porosity and acidity of soil and rocks, total dissolved solids, and alkalinity [7]. Thus, high variations in fluoride levels can be observed: while in some parts of the world the naturally occurring fluoride concentration exceeds the threshold value of 1.5 mg/L as established by the WHO, other areas possess drinking water with fluoride levels below their recommended minimal concentration of 0.5 mg/L for preventing dental caries [8]. According to the United States Public Health Service, the ideal level to balance the benefits of fluorides on caries prevention while limiting harmful health effects is 0.7 mg/L [9]. In some countries, artificial fluoridation of water supplies is performed to achieve optimal fluoride levels. In Europe, however, community water fluoridation is rejected by most countries, including Austria [10]. In 1993, a nationwide analysis of the fluoride concentration in Austrian drinking water was conducted, revealing insufficient levels in almost all regions, which has also been observed in neighboring countries [11,12,13]. The last nationwide fluoride analysis of drinking water samples, which was conducted in 2007, not only reconfirmed the findings but also showed an even further decrease in the fluoride levels. At this time, a fluoride concentration higher than 0.5 mg/L has only been observed in the water samples of 30 municipalities [14].

It is important for dental professionals to consider local variations in the fluoride concentration in drinking water to provide adequate recommendations regarding topical/systemic fluoride application, and to avoid over-/underdosing. Therefore, the aim of the present study was to re-examine the fluoride concentration in Austrian drinking water nationwide.

## 2. Materials and Methods

Drinking water samples were collected in a total of 1985 municipalities in Austria. In each location, drinking water was collected within one day from three different sources, which were randomly chosen (private households, public restrooms, restaurants, shops, gas stations, etc.). After letting the water run for 30 s to avoid contamination, 500 mL of drinking water was collected in fresh 500 mL high-density polypropylene bottles (Flaschenland, Ransbach-Baumbach, Germany). Samples were stored at room temperature for a maximum of 14 days before analysis.

The fluoride concentration in the collected drinking water samples was analyzed in the Department of Analytical Chemistry, Faculty of Chemistry, University of Vienna, using a fluoride-ion-selective electrode (6.0502.150; Metrohm AG, Herisau, Switzerland), a Ag/AgCl reference electrode (3 M KCl, 6.0726.100, Metrohm AG, Herisau, Switzerland), and a 691 voltmeter (8.691.1001; Metrohm AG, Herisau, Switzerland). To calibrate the device and for further regression analysis, fluoride standard solutions were prepared with the following concentrations: 0.05, 0.1, 0.2, 0.5, 1, 5, and 10 mg/L. The detected voltage was plotted against the standard concentration for quantitative analysis via external calibration. From each sample, 50 mL of drinking water was mixed with 5 mL of the total ionic strength adjustment buffer, which regulates the ionic strength and pH of the sample. One liter of the TISAB II buffer contains 58 g NaCl, 3.0 g sodium citrate dihydrate, 57 mL acetic acid (100%), and pure water. Samples and standard solutions were stored for 24 h to achieve an equal temperature of 20 °C prior to analysis. Each sample was analyzed twice, and mean values were determined.

Data management, descriptive, and inferential statistics were conducted with Microsoft Excel 2016 (Microsoft Inc., Redmond, WA, USA) and GraphPad Prism 10 (GraphPad Software, Boston, MA, USA). Differences between fluoride level mean values in drinking water of the different regions were assessed by the ANOVA statistic with the Bonferroni post hoc test. The correlation between fluoride level and the caries prevalence was analyzed by the Spearman Rho method. The statistical analysis was performed with SPSS 27.0 (IBM, Armonk, NY, USA).

## 3. Results

### 3.1. Municipalities Included in the Study

Austria is structured into nine different regions, with Lower Austria having the largest area and Vienna having the smallest area. These regions are divided into a total of 2092 municipalities. As shown in Table 1, drinking water samples were collected in 1985 from 2092 Austrian municipalities.

### 3.2. Fluoride Concentration in Austrian Drinking Water

**Figure 1:**
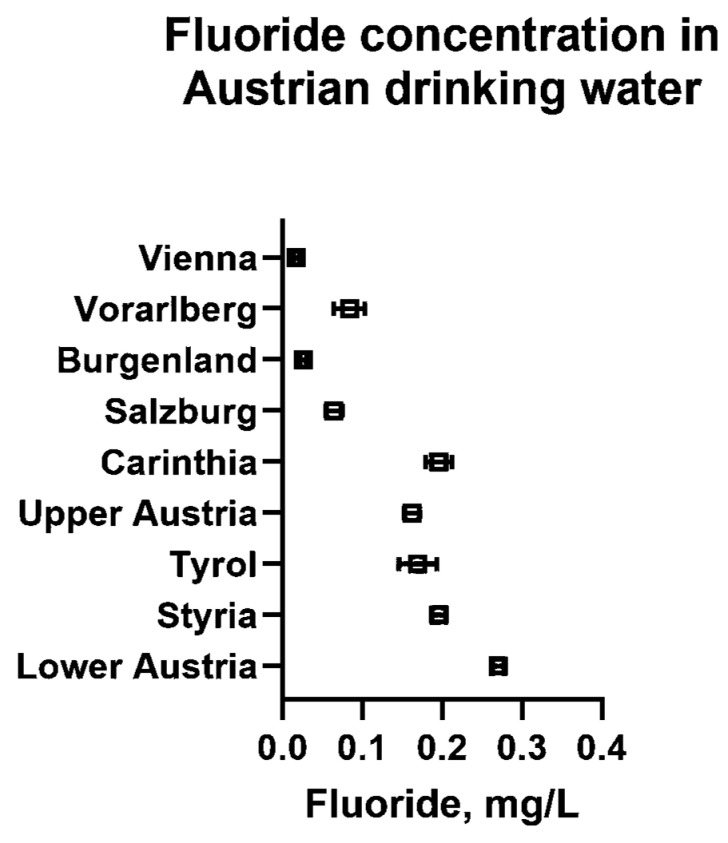
Mean fluoride concentration in Austrian drinking water per region. Data represent the mean values and 95% confidence intervals. Appendix A Table A1 demonstrates the analysis of the differences in fluoride concentrations in the drinking water between the nine Austrian regions.

**Figure 2:**
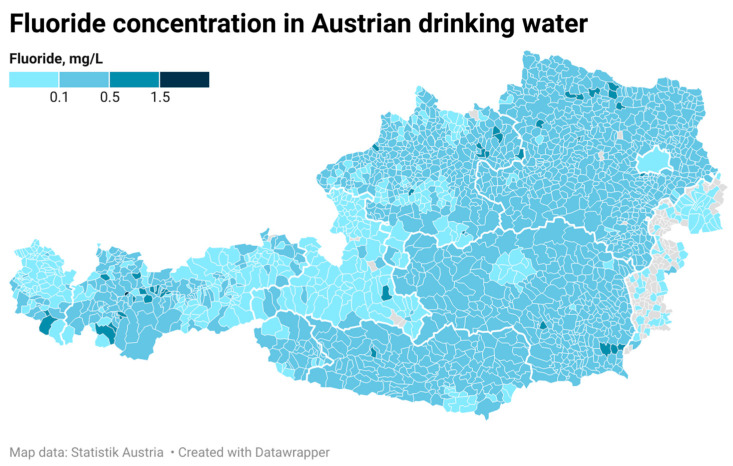
Regional variations in fluoride levels in the drinking water of Austrian municipalities.

Figure 1 shows the mean fluoride concentration with 95% CI found in the drinking water of all nine Austrian regions. While Lower Austria had the highest mean fluoride concentration with 0.27 mg/L, the lowest values were observed in Vienna, Vorarlberg, Burgenland, and Salzburg, with average values below the detection limit of 0.1 mg/L. The values reached neither the optimal level of 0.7 mg/L established by the United States Public Health Service, nor the minimal concentration for carioprotective effects (0.5 mg/L) indicated by the WHO [7,8]. The regional variations in fluoride concentration in the drinking water of Austrian municipalities are demonstrated in Figure 2.

**Figure 3:**
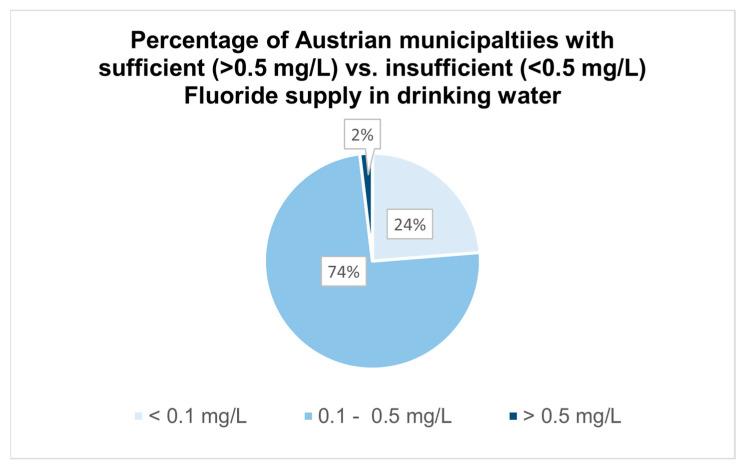
Percentage shares of Austrian municipalities with sufficient vs. insufficient fluoride concentration in drinking water.

In total, only 38 of the 1985 included municipalities exceeded the minimal concentration of 0.5 mg/L, which represents 2% of all measured locations, as shown in Figure 3. Out of those municipalities, 16 locations showed fluoride levels above 0.7 mg/L, one of them (Karrösten, Tyrol) even exceeding the WHO threshold value with a concentration of 1.92 mg/L. Most of the municipalities, precisely 1505 (74%), exhibited drinking water with a fluoride concentration of 0.1–0.5 mg/L. Almost one quarter of the drinking water samples showed fluoride levels below 0.1 mg/L.

**Figure 4:**
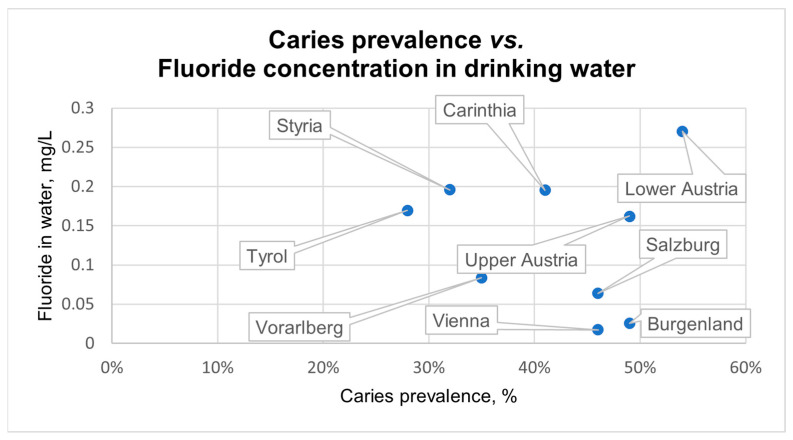
Relationship between caries prevalence of 6–7-year-old children and fluoride concentration in the drinking water of nine Austrian regions. A very weak, non-significant negative correlation between these parameters was observed (Spearman-Rho r = −0.126, *p* = 747).

Figure 4 demonstrates the relationship between available caries prevalence data (children aged 6–7 years) and the fluoride concentration in the drinking water of nine Austrian regions [2]. Although a weak inverse relationship between these parameters can be observed, the analysis revealed no statistically significant correlation.

As presented in Table 2, the fluoride concentrations measured in 2007 and 2023 showed some relevant differences. The table contains all municipalities that showed fluoride levels above 0.5 mg/L in 2007, which was observed in 30 locations). In the recent analysis, 24 of these 30 municipalities had a lower fluoride concentration in their drinking water, 16 of them being below 0.5 mg/L. Out of the six municipalities with higher values in comparison to the assessment 15 years ago, one clearly exceeded the WHO guideline value of 1.5 mg/L.

Appendix A Table A1 demonstrates the analysis of the differences in fluoride concentrations in the drinking water between the nine Austrian regions, showing significant differences between numerous areas (*p* > 0.05).The exact fluoride levels in the drinking water of all 1985 municipalities are provided in Appendix A Table A2.

## 4. Discussion

Fluoride, the anion of the trace element fluorine, naturally occurs in water, air, and soil. Due to its chemical reactivity potential, fluorine is often bound to other chemical elements, e.g., metals of the first and second columns of the periodic table [15]. The amount of minerals and their solubility in drinking water primarily depend on the local, regional, and geological settings, as well as the hydro-geological conditions [16]. Austria has at least four different mountain/rock types, which contribute to the variety of fluoride concentrations found throughout the country [17,18]. While the fluoride concentration in the drinking water of the regions Vienna, Vorarlberg, Burgenland, and Salzburg was mostly below the detection limit, higher levels have been observed in areas like Lower Austria, Carinthia, Styria, and Tyrol. Similar tendencies have been observed in the analyses conducted in 1993 and 2007.

Overall, the drinking water of most municipalities analyzed in our study showed fluoride levels below 0.5 mg/L (98%), which is in accordance with the findings from the nationwide analyses in 1993 and 2007 [11,12,14]. Comparably low fluoride concentrations in drinking water have been observed in Germany. As reported by the German Federal Institute for Risk Assessment in 2025, 90% of German drinking water samples contain less than 0.3 mg/L of fluoride. A nationwide investigation from 2003 revealed similar results in Switzerland, showing fluoride levels below 0.3 mg/L in most cantons [13].

Low fluoride concentrations in drinking water could constitute an additional risk factor for developing caries, especially for individuals with a high susceptibility to dental cavities [19]. Caries is a widespread disease caused by a disbalance in the oral microbiome, a disturbance in the process of demineralization and remineralization, as well as a specific host response [20,21]. Since 2006, the Austrian National Public Health Institute (GÖG) has regularly assessed the dental status of Austrian 6- to 7-year-olds in some Austrian regions. The highest rates of children with caries experience were observed in Salzburg (2006: 69%) and Vienna (2006: 66%). Starting from 2016, the GÖG performed their evaluations in all Austrian regions, constituting the only source of data capturing caries prevalence nationwide. Comparing the analyses of 2016 and 2024, the proportion of Austrian 6- to 7-year-old children with caries experience slightly improved from an average of 45% to 42%. The lowest rates of children affected by dental caries were reported in Tyrol (2016: 30%; 2024: 28%), followed by Styria (2016: 34%; 2024: 32%). Interestingly, they are among the regions with higher fluoride levels in drinking water. Although the analysis revealed a weak tendency, no statistically significant correlation between caries prevalence and fluoride concentration in drinking water has been observed. This might be explained by the multitude of factors influencing caries prevalence, as shown by the GÖG, including sociodemographic factors. Although there was a major improvement in caries prevalence since 2006, the WHO goal of 80% caries-free children, or rather a maximum prevalence of 20%, is yet to be reached [2].

Apart from adequate oral hygiene and dietary measures, fluorides are considered one of the most important preventive actions against caries [22,23]. Their effectiveness is based on several different mechanisms: the presence of fluoride in dental plaque and saliva increases the remineralization of incipient enamel lesions and prevents demineralization. In an acidic environment, fluoride penetrates the enamel subsurface and forms crystals related to those of fluorapatite, which protects the enamel from dissolving. Furthermore, they interfere with the glycolysis of cariogenic bacteria and exhibit bactericidal effects in higher concentrations. Although the method of action is predominantly based on post-eruptive and topical effects, fluorides might increase the resistance of teeth against caries when ingested during tooth development. Apart from that, systemic fluoride intake also has topical effects by increasing the salivary fluoride concentration [24,25,26,27]. Accordingly, a substantial decrease in caries prevalence has been reported in areas with optimally fluoridated drinking water [28].

The beneficial effects of fluorides in proper amounts are undisputable; however, the total intake (topical and systemic) should be monitored to prevent overdosage [24]. As recommended by the WHO, the fluoride concentration in drinking water should not exceed the guideline value of 1.5 mg/L. Yet, the drinking water in the Tyrolean municipality of Karrösten contained 1.98 mg/L of fluorides, which might have potential adverse effects. At concentrations of 0.9–1.2 mg/L, mild dental fluorosis might occur, depending on the amount of other fluoride exposure sources [8]. These lesions are caused by long-term ingestion of higher fluoride levels during tooth mineralization and can range from small, white, almost unremarkable spots up to easily visible, large white areas of the enamel [26]. The only publication concerning dental fluorosis prevalence in Austria dates back to 1961, assessing 400 children aged 6 to 14 years old who were living in Mallnitz and other locations in the Ötz Valley with fluoride levels over 1.0 mg/L. In locations with 1.0 mg/L, the prevalence of fluorosis was 18.7%, reaching up to 59% in a municipality with 1.8 mg/L of fluoride in drinking water [29]. More recent data are available from the USA, where dental fluorosis prevalence has been repeatedly assessed for almost 40 years. While the prevalence has gradually increased from 22% in 1986/1987 to 87.3% in 2013/2014, it dropped to 68.2% in 2015/2016, possibly due to recommendations by the Department of Health and Human Services to lower water fluoridation in 2015 [30,31]. In contrast, a systematic review including international data revealed dental fluorosis prevalence of 40% at fluoride concentrations of 0.7 mg/L, although only 12% had aesthetic concerns [32]. However, all these studies suggest that higher fluoride levels are associated with a greater risk of dental fluorosis.

Higher levels of fluoride around 3–6 mg/L can lead to skeletal fluorosis, and at levels above 10 mg/L, a notably higher risk of bone fractures is described [8]. Prevalence data for skeletal fluorosis are not available for Europe and the USA, which might be due to the limited occurrence. Interestingly, a recent meta-analysis revealed an increased risk of skeletal fluorosis even at fluoride levels within the national guidelines [33]. In Austria, fluoride overdosage, especially intoxication, is highly unlikely, as long as the use of other fluoride sources is not exaggerated [12].

The present data could support dental professionals in individually advising their patients on the use of fluorides, depending on their place of residence. However, the present study is limited by possible seasonal and geological variations. Due to the dynamics of the fluoride concentrations throughout the years (as demonstrated in Table 2), measurements should be performed in regular intervals throughout the year to provide updated values. In addition, fluoride analyses of drinking water might be used as a basis for guidelines and recommendations by the Austrian health care system or the government for specific areas. These adjustments could reduce the need for restorative procedures in the dental office and, therefore, decrease the costs of the Austrian health care system. Furthermore, this may support the achievement of a paradigm shift from a reactive to an active, and thus preventive, strategy.

## 5. Conclusions

The present study revealed that the fluoride concentration in Austrian drinking water is below international recommendations for protective effects against dental caries in almost all municipalities, confirming the findings from 1993 and 2007. However, the fluoride levels in the drinking water of one Tyrolian municipality exceeded the WHO threshold value. These data should be taken into consideration for individual caries risk evaluation and may serve as a basis for targeted recommendations regarding fluoride supplementation.

## Figures and Tables

**Table 1:** Number of municipalities per region included in this study.

Province	Municipalities *
Lower Austria	573
Styria	286
Tyrol	277
Upper Austria	438
Carinthia	132
Salzburg	119
Burgenland	61
Vorarlberg	96
Vienna	3
**TOTAL**	**1985**

* included in the study (1985 out of 2092 municipalities).

**Table 2:** Changes in fluoride concentrations in the drinking water of municipalities with levels > 0.5 mg/L in 2007.

	2007	2023	↑/↓		2007	2023	↑/↓
**Vorarlberg**				**Lower Austria**			
Innerbraz	0.65	0.33	↓	Payerbach	0.57	0.29	↓
St. Anton	0.51	0.35	↓	St. Georgen am Ybbsfelde	1.14	0.32	↓
St. Gallenkirch	0.64	0.51	↓	**Salzburg**			
Schruns	0.7	0.54	↓	Bad Gastein	2.35	0.17	↓
**Tyrol**				**Carinthia**			
Biberwier	0.88	1.26	↑	Mallnitz	0.53	0.20	↓
Elmen	0.53	0.99	↑	Oberdrauburg	0.59	0.30	↓
Faggen	0.53	0.34	↓	**Upper Austria**			
Haiming	1.37	<0.01	↓	Bad Zell	2.09	0.14	↓
Inzing	0.66	1.09	↑	Hargelsberg	0.92	0.11	↓
Karrösten	1.58	1.92	↑	Mehrnbach	0.95	0.16	↓
Kaunerberg	1.3	0.93	↓	Münzbach	1.24	0.39	↓
Oberhofen	0.5	0.61	↑	Ottenschlag im Mühlkreis	0.57	0.12	↓
Rietz	0.92	1.00	↑	Ried in der Riedmark	0.51	0.5	↓
Silz	1.71	0.85	↓	Schönau im Mühlkreis	0.63	0.59	↓
St. Leonhard	1.08	0.21	↓	Windhaag bei Perg	1.17	0.42	↓
Tösens	0.6	0.5	↓	Windischgarsten	0.69	0.57	↓

↑ Fluoride concentration in 2023 is higher than in 2007; ↓ Fluoride concentration in 2023 is lower than in 2007.

## Data Availability

The original contributions presented in this study are included in the article. Further inquiries can be directed to the corresponding author.

## Abbreviations

The following abbreviations are used in this manuscript:

CI	Confidence Interval
WHO	World Health Organization

## Appendix A

### Appendix A.1

**Table A1:** Statistical analysis of the differences in fluoride concentration currently measured in the drinking water between various regions of Austria. The differences were analyzed using the ANOVA statistic with a Bonferroni post hoc test. The *p*-values originated from this test are presented. Differences with *p* < 0.05 were considered statistically significant and are marked in bold.

	Lower Austria	Styria	Tyrol	Upper Austria	Carinthia	Salzburg	Burgenland	Vorarlberg	Vienna
Lower Austria		0.000	0.000	0.000	0.000	0.000	0.000	0.000	0.003
Styria	0.000		0.151	0.002	1.000	0.000	0.000	0.000	0.185
Tyrol	0.000	0.151		1.000	0.848	0.000	0.000	0.000	0.620
Upper Austria	0.000	0.002	1.000		0.076	0.000	0.000	0.000	0.829
Carinthia	0.000	1.000	0.848	0.076		0.000	0.000	0.000	0.197
Salzburg	0.000	0.000	0.000	0.000	0.000		0.931	1.000	1.000
Burgenland	0.000	0.000	0.000	0.000	0.000	0.931		0.047	1.000
Vorarlberg	0.000	0.000	0.000	0.000	0.000	1.000	0.047		1.000
Vienna	0.003	0.185	0.620	0.829	0.197	1.000	1.000	1.000	

### Appendix A.2

**Table A2:** Currently measured fluoride concentration in the drinking water of Austrian municipalities (in total 1985 locations).

Fluoride Concentration in Austrian Drinking Water (mg/L)					
Lower Austria					
Absdorf	0.46	Hernstein	0.29	Raabs an der Thaya	0.26
Achau	0.41	Herrnbaumgarten	0.25	Raach am Hochgebirge	0.28
Aderklaa	0.26	Herzogenburg	0.33	Raasdorf	0.17
Aggsbach	0.42	Himberg	0.19	Rabensburg	0.22
Alberndorf im Pulkautal	0.47	Hinterbrühl	0.34	Rabenstein an der Pielach	0.27
Albrechtsberg an der Gr. Krems	0.28	Hirschbach	0.22	Ramsau	0.26
Alland	0.23	Hirtenberg	0.3	Randegg	0.25
Allensteig	0.27	Hochleithen	0.3	Rappottenstein	0.21
Allhartsberg	0.2	Hochneukirchen-Gschaidt	0.25	Rastenfeld	0.26
Altenburg	0.28	Hochwolkersdorf	0.29	Rauchenwarth	0.2
Altendorf	0.23	Hof am Leithagebirge	0.23	Ravelsbach	0.45
Altenmarkt an der Triesting	0.22	Hofamt Priel	0.22	Raxendorf	0.27
Altlengbach	0.29	Höflein	0.28	Reichenau an der Rax	0.29
Altlichtenwarth	0.23	Höflein an der Hohen Wand	0.25	Reinigers	0.28
Altmelon	0.27	Hofstetten-Grünau	0.27	Reinsberg	0.29
Amaliendorf-Aalfang	0.23	Hohe Wand	0.28	Reisenberg	0.26
Amstetten	<0.1	Hohenau an der March	0.28	Retz	0.77
Andlersdorf	0.28	Hohenberg	0.23	Retzbach	0.29
Angern an der March	0.27	Hohenreich	0.27	Ringelsdorf	0.28
Annaberg	0.23	Hohenruppersdorf	0.21	Rohr im Gebirge	0.26
Arbesbach	0.28	Hohenwath-Mühlbach am Manhartsb.	0.28	Rohrau	0.3
Ardagger	0.2	Hollabrunn	0.32	Rohrbach an der Gölsen	0.47
Artstetten-Pöbring	0.18	Hollenstein an der Ybbs	0.18	Rohrdorf bei Krems	0.31
Aschbach-Markt	0.19	Hollenthon	0.28	Röhrenbach	0.32
Aspangberg-St. Peter	0.28	Horn	0.41	Röschitz	0.27
Aspang-Markt	0.28	Hundesheim	0.29	Rosenburg-Mold	0.48
Asparn an der Zaya	0.33	Hürm	0.27	Rossatz-Arnsdorf	0.27
Asperhofen	0.27	Inzersdorf-Getzersdorf	0.33	Ruprechtshofen	0.14
Atzenbrugg	0.26	Irnfritz-Messern	0.36	Rußbach	0.27
Au am Leithaberge	0.26	Jaidhof	0.24	Sallingberg	0.28
Auersthal	0.26	Japons	0.3	Sankt Martin-Karlsbach	0.2
Bad Deutsch-Altenburg	0.3	Jedenspeigen	0.23	Sankt Oswald	0.25
Bad Erlach	0.25	Judenau-Baumgarten	0.24	Scharndorf	0.29
Bad Fischau-Brunn	0.31	Kaltenleutgeben	0.35	Scheibbs	0.22
Bad Großpertholz	0.27	Kapelln	0.36	Scheiblingkirchen-Thernberg	0.27
Bad Pirawarth	0.28	Karlstein an der Thaya	0.26	Schollach	0.25
Bad Schönau	0.28	Karlstetten	0.27	Schönau an der Triesting	0.29
Bad Traunstein	0.27	Kasten bei Böheimkirchen	0.28	Schönbach	0.26
Bad Vöslau	0.23	Katzelsdorf	0.29	Schönberg am Kamp	0.4
Baden bei Wien	0.23	Kaumberg	0.23	Schönbühel-Aggsbach	0.2
Bärnkopf	0.2	Kautzen	0.28	Schönkirchen-Reyersdorf	0.33
Behamberg	0.15	Kematen an der Ybbs	0.2	Schottwien	0.24
Berg	0.23	Kilb	0.3	Schrattenbach	0.28
Bergern im Dunkelsteinerwald	0.25	Kirchberg am Walde	0.27	Schrattenberg	0.32
Bergland	0.21	Kirchberg am Wechsel	0.28	Schrattenthal	0.44
Berndorf	0.28	Kirchberg an der Pielach	0.28	Schrems	0.27
Bernhardsthal	0.28	Kirchschlag	0.14	Schwadorf	0.26
Biberbach	0.12	Kirchschlag in der Buckligen Welt	0.27	Schwarzau im Gebirge	0.24
Biedermannsdorf	0.29	Kirchstetten	0.31	Schwarzenau	0.23
Bisamberg	0.25	Kirnberg an der Mank	0.23	Schwarzenbach	0.27
Bischofstetten	0.25	Klausen-Leopoldsdorf	0.28	Schwarzenbach an der Pielach	0.27
Blindenmarkt	0.25	Klein-Neusiedl	0.3	Schwazau am Steinfeld	0.23
Blumau-Neurißhof	0.31	Klein-Pöchlarn	0.26	Schwechat	0.17
Bockfließ	0.27	Kleinzell	0.24	Schweiggers	0.16
Böheimkirchen	0.28	Klosterneuburg	0.25	Seebenstein	0.24
Brand-Laaben	0.27	Königsbrunn am Wagram	0.3	Seefeld-Kadolz	0.27
Brand-Nagelberg	0.27	Königstetten	0.29	Seibersdorf	0.32
Breitenau	0.26	Korneuburg	0.28	Seitenstetten	0.13
Breitenfurt bei Wien	0.32	Kottes-Purk	0.28	Semmering	0.26
Breitenstein	0.29	Kottingbrunn	0.21	Senftenberg	0.25
Bromberg	0.25	Krems an der Donau	0.33	Setteldorf am Wagram	0.29
Bruck an der Leitha	0.44	Kreutal	0.29	Sieghartskirchen	0.24
Brunn am Gebirge	0.31	Kreuzstetten	0.27	Sierndorf	0.42
Brunn an der Wild	0.57	Krichberg am Wagram	0.3	Sigmundsherberg	0.81
Buchbach	0.26	Krumau am Kamp	0.14	Sitzenberg-Reidling	0.29
Burgschleinitz-Kühnring	0.28	Krumbach	0.29	Sitzendorf an der Schmida	0.28
Burg-Vöstenhof	0.26	Krummnußbaum	0.23	Sollenau	0.25
Deutsch-Wagram	0.38	Laa an der Thaya	0.28	Sommerrein	0.25
Dietmanns	0.46	Laab im Walde	0.18	Sonntagberg	0.13
Dobersberg	0.25	Labendorf	0.25	Sooß	0.33
Dorfstetten	0.25	Langau	0.49	Spannberg	0.28
Drasenhofen	0.29	Langenlois	0.39	Spillern	0.28
Drosendorf-Zisserdorf	0.3	Langenrohr	0.26	Spitz	0.38
Drösing	0.26	Langenzersdorf	0.11	St Pölten	0.25
Droß	0.19	Langschlag	0.29	St. Aegyd am Neuwalde	0.28
Dunkelsteinerwald	0.21	Lanzendorf	0.29	St. Andrä-Wördern	0.25
Dürnkrut	0.21	Lanzenkirchen	0.29	St. Anton an der Jeßnitz	0.27
Dürnstein	0.39	Lassee	0.16	St. Bernhard-Frauenhof	0.58
Ebenfurth	0.28	Laxenburg	0.28	St. Corona am Wechsel	0.27
Ebenthal	0.28	Leiben	0.25	St. Egyden am Steinfeld	0.29
Ebergassing	0.16	Leitzersdorf	0.18	St. Georgen am Reith	0.12
Ebreichsdorf	0.3	Lengfelden	0.16	St. Georgen am Ybbsfelde	0.32
Echsenbach	0.26	Leobendorf	0.27	St. Georgen an der Leys	0.26
Eckartsau	0.26	Leobersdorf	0.22	St. Leonhard am Forst	0.18
Edlitz	0.25	Leopoldsdorf	0.13	St. Leonhard am Hornerwald	0.32
Eggenburg	0.27	Leopoldsdorf im Marchfelde	0.18	St. Margaretehn an der Siering	0.25
Eggendorf	0.28	Lichtenau im Waldviertel	0.3	St. Martin	0.27
Eggern	0.2	Lichtenegg	0.28	St. Pantaleon-Erla	0.15
Eichgraben	0.29	Lichtenwörth	0.26	St. Peter in der Au	0.17
Eisgarn	0.19	Lilienfeld	0.26	St. Valentin	0.21
Emmersdorf an der Donau	0.16	Litschau	0.24	St. Veit an der Gölsen	0.25
Engelhartstetten	0.2	Loich	0.26	Staatz	0.24
Ennsdorf	0.16	Loosdorf	0.27	Statzendorf	0.28
Enzenreith	0.28	Ludweis-Aigen	0.25	Steinakirchen am Forst	0.25
Enzersdorf an der Fischa	0.21	Lunz am See	0.24	Stetten	0.32
Enzersfeld	0.36	Mailberg	0.26	Stockerau	0.3
Enzesfeld-Lindabrunn	0.21	Maissau	0.31	Stössing	0.26
Erlauf	0.21	Mank	0.13	Straning-Grafenberg	0.31
Ernstbrunn	0.26	Mannersdorf am Leithagebirge	0.31	Straß im Straßertale	0.27
Ernsthofen	0.18	Mannsdorf an der Donau	0.35	Strasshof an der Nordbahn	0.25
Ertl	0.14	Marbach an der Donau	0.24	Stratzing	0.29
Eschenau	0.24	Marchegg	0.4	Strengberg	0.12
Euratsfeld	0.15	Maria Anzbach	0.32	Stronsdorf	0.23
Falkenstein	0.28	Maria Enzersdorf	0.17	Sulz im Weinviertel	0.27
Fallbach	0.26	Maria Laach am Jauerling	0.27	Tattendorf	0.28
Feistritz am Wechsel	0.24	Maria Taferl	0.26	Teesdorf	0.29
Felixdorf	0.32	Maria-Lanzendorf	0.16	Ternitz	0.28
Fels am Wagram	0.44	Markersdorf-Haindorf	0.29	Texingtal	0.27
Ferschnitz	0.21	Markgrafneusiedl	0.16	Thaya	0.27
Fischamed	0.28	Markt Piesting	0.33	Theresienfeld	0.29
Frankenfels	0.27	Martinsberg	<0.1	Thomasberg	0.26
Furth an der Triesting	0.27	Matzendorf-Hölles	0.27	Traisen	0.31
Furth bei Göttweig	0.29	Matzen-Raggendorf	0.21	Traiskirchen	0.23
Gaaden	0.31	Mauerbach	0.33	Traismauer	0.33
Gablitz	0.42	Mautern an der Donau	0.73	Trattenbach	0.29
Gaming	0.27	Meisldorf	0.3	Trautmannsdorf an der Leitha	0.22
Gänserndorf	0.33	Melk	0.2	Trumau	0.24
Gars am Kamp	0.42	Michelbach	0.3	Tulbing	0.31
Gastern	0.28	Michelhausen	0.28	Tulln an der Donau	0.38
Gaubitsch	0.25	Miesenbach	0.27	Tullnerbach	0.11
Gaweinstal	0.34	Mistelbach an der Zaya	0.33	Türnitz	0.25
Gedersdorf	0.25	Mitterbach am Erlaufsee	0.14	Ulrichskirchen-Schleinbach	0.28
Geras	0.32	Mitterndorf an der Fischa	0.28	Unserfrau-Altweira	0.23
Gerasdorf bei Wien	0.15	Mödling	0.31	Untersiebenbrunn	0.26
Gerersdorf	0.29	Mönichkirchen	0.27	Unterstinkenbrunn	0.28
Gföhl	0.3	Moorbad Harbach	0.27	Velm-Götzendorf	0.24
Gießhübl	0.33	Moosbrunn	0.22	Viehdorf	0.16
Glinzendorf	0.28	Muckendorf-Wipfing	0.27	Vitis	0.29
Gloggnitz	0.27	Muggendorf	0.29	Vösendorf	0.29
Gmünd	0.28	Mühldorf	0.3	Waidhofen an der Thaya	0.25
Gnadendorf	0.2	Münchendorf	0.26	Waidhofen an der Thaya-Land	0.24
Göllersdorf	0.27	Münichreith-Laimbach	0.28	Waidhofen an der Ybbs	0.23
Golling an der Erlauf	0.26	Nappersdorf-Kammersdorf	0.28	Waidmannsfeld	0.28
Göpfritz an der Wild	0.29	Natschbach-Loipersbach	0.29	Waldegg	0.25
Göstling an der Ybbs	0.22	Neidling	0.26	Waldenstein	0.28
Göttlesbrunn-Arbesthal	0.28	Neudorf im Weinviertel	0.28	Waldhausen	0.16
Götzendorf an der Leitha	0.19	Neuhofen an der Ybbs	0.2	Waldkirchen an der Thaya	0.22
Grabern	0.79	Neulengbach	0.27	Wallsee Sindelburg	0.14
Grafenbach-St. Valentin	0.28	Neumarkt an der Ybbs	0.3	Walpersbach	0.28
Grafenegg	0.31	Neunkrichen	0.26	Wang	0.23
Grafenschlag	0.7	Neusiedl an der Zaya	0.23	Warth	0.26
Grafenwörth	0.43	Neustadl an der Donau	0.21	Wartmannstetten	0.21
Gramatneusiedl	0.3	Neustift-Innermanzing	0.26	Weiden an der March	0.2
Gresten	0.29	Niederabsdorf	0.28	Weikendorf	0.28
Gresten-Land	0.29	Niederhollabrunn	0.26	Weikersdorf am Steinefelde	0.26
Grimmenstein	0.28	Niederleis	0.26	Weinburg	0.23
Groß Gerungs	0.21	Nöchling	0.26	Weinzierl am Walde	0.26
Großdietmanns	0.23	Nußdorf ob der Traisen	0.33	Weissenbach an der Triesting	0.28
Großebersdorf	0.26	Ober-Grafendorf	0.28	Weißenkirchen a. d. Perschling	0.26
Großengersdorf	0.28	Oberndorf an der Melk	0.24	Weißenkirchen in der Wachau	0.3
Groß-Enzersdorf	0.2	Obersiebenbrunn	0.27	Weistrach	0.15
Großgöttfritz	0.15	Oberwalterdorf	0.26	Weiten	0.25
Großharras	0.26	Obritzberg-Rust	0.28	Weitersfeld	0.28
Großhofen	0.17	Oed-Oehling	0.17	Weitra	0.28
Großkrut	0.28	Opponitz	0.18	Wiener Neudorf	0.28
Großmugl	0.28	Orth an der Donau	0.33	Wiener Neustadt	0.3
Großriedenthal	0.27	Ottenschlag	0.14	Wienerwald	0.28
Großrußbach	0.27	Ottenthal	0.26	Wieselburg	0.28
Großschönau	0.21	Otterthal	0.25	Wieselburg-Land	0.26
Groß-Schweinbarth	0.27	Paltendorf-Dobermannsdorf	0.28	Wiesmath	0.23
Groß-Siegharts	0.25	Parbasdorf	0.28	Wildendürnbach	0.28
Großweikersdorf	0.25	Paudorf	0.27	Wilfersdorf	0.38
Grünbach am Schneeberg	0.28	Payerbach	0.29	Wilhelmsburg	0.37
Gumpoldskirchen	0.23	Perchtoldsdorf	0.59	Willendorf	0.28
Günseldorf	0.22	Pernegg	0.3	Wimpassing im Schwarzatale	0.25
Guntersdorf	0.43	Pernersdorf	0.28	Windigsteig	0.25
Guntramsdorf	0.3	Pernitz	0.21	Winklarn	0.18
Gutenbrunn	0.22	Persenbeug-Gottsdorf	0.28	Winzendorf-Muthmannsdorf	0.14
Gutenstein	0.25	Petronell-Carnuntum	0.33	Wölbing	0.29
Haag	0.21	Petzenkirchen	0.24	Wolfsbach	0.18
Hadersdorf-Kammern	0.25	Pfaffenschlag bei Waidhofen/Thaya	0.24	Wolfsgraben	0.1
Hadres	0.39	Pfaffstätten	0.3	Wolfspassing	0.28
Hafnerbach	0.26	Pillichsdorf	0.24	Wolfsthal	0.24
Hagenbrunn	0.38	Pitten	0.26	Wolkersdorf im Weinviertel	0.28
Haidershofen	0.13	Pöchlarn	0.12	Wöllersdorf-Steinabrückl	0.21
Hainburg an der Donau	0.37	Pöggstall	0.3	Wullersdorf	0.28
Hainfeld	0.33	Pölla	0.37	Würmla	0.23
Hardegg	0.46	Pottendorf	0.28	Ybbs an der Donau	0.16
Haringsee	0.24	Pottenstein	0.29	Ybbsitz	0.19
Harmannsdorf	0.26	Poysdorf	0.39	Yspertal	0.27
Haslau-Maria Ellend	0.22	Prellenkirchen	0.32	Zeillern	0.23
Haugschlag	0.28	Pressbaum	0.1	Zeiselmauer-Wolfpassing	0.26
Haugsdorf	0.36	Prigglitz	0.26	Zelking-Matzleinsdorf	0.17
Haunoldstein	0.29	Prinzersdorf	0.29	Zellerndorf	0.51
Hausbrunn	0.26	Prottes	0.24	Ziersdorf	0.29
Hauskirchen	0.13	Puchberg am Schneeberg	0.28	Zillingdorf	0.28
Hausleiten	0.24	Puchenstuben	0.27	Zistersdorf	0.32
Heidenreichstein	0.27	Pulkau	0.38	Zöbern	0.29
Heiligenkreuz	0.27	Purgstall an der Erlauf	0.28	Zwentendorf an der Donau	0.28
Heldenberg	0.28	Purkersdorf	0.34	Zwettl	0.28
Hennersdorf	0.29	Pyhra	0.28	Zwölfaxing	<0.1
**Styria**					
Admont	0.19	Kainach bei Voitsberg	0.25	Schöder	0.25
Aflenz	0.11	Kainbach bei Graz	0.23	Schwarzautal	0.24
Aich	0.29	Kaindorf	0.31	Seckau	0.12
Aigen im Ennstal	0.41	Kalsdorf bei Graz	0.14	Seiersberg-Pirka	0.14
Albersdorf-Prebuch	0.18	Kalwang	<0.1	Selzthal	0.18
Allerheiligen bei Wildon	0.27	Kammern bei Liesingtal	<0.1	Semriach	0.23
Altaussee	0.13	Kapfenberg	0.23	Sinabelkirchen	0.15
Altenmarkt bei St. Gallen	0.11	Kapfenstein	0.52	Söchau	0.19
Anger	0.15	Kindberg	0.28	Söding-St. Johann	0.19
Ardning	0.19	Kirchbach-Zerlach	0.24	Sölk	0.14
Arnfels	0.24	Kirchberg an der Raab	0.19	Spielberg	0.15
Bad Aussee	0.14	Kitzeck im Sausal	0.23	Spital am Semmering	0.12
Bad Blumau	0.2	Klöch	0.22	St. Andrä-Höch	0.25
Bad Gleichenberg	0.51	Knittelfeld	0.12	St. Anna am Aigen	0.22
Bad Loipersdorf	0.18	Kobenz	0.14	St. Barbara im Mürztal	0.19
Bad Mitterndorf	0.11	Köflach	0.14	St. Bartholomä	0.16
Bad Radkersburg	0.2	Krakau	0.19	St. Gallen	0.1
Bad Schwanberg	0.26	Kraubath an der Mur	<0.1	St. Georgen am Kreischberg	0.19
Bad Waltersdorf	0.34	Krieglach	0.19	St. Georgen an der Stiefing	0.23
Bärnbach	0.18	Krottendorf-Gaisfeld	0.34	St. Georgen ob Judenburg	0.24
Birkfeld	0.13	Kumberg	0.12	St. Jakob im Walde	0.14
Breitenau am Hochlantsch	0.16	Lafnitz	0.18	St. Johann im Saggautal	0.23
Bruck an der Mur	0.15	Landl	0.12	St. Johann in der Haide	0.34
Buch-St. Magdalena	0.35	Lang	0.17	St. Josef (Weststeiermark)	0.2
Burgau	0.2	Langenwang	0.2	St. Katherein am Hauenstein	0.14
Dechantskirchen	0.15	Lannach	0.2	St. Katherein am Offenegg	0.17
Deutsch Goritz	0.24	Lassing	0.3	St. Lambrecht	0.18
Deutschfeistritz	0.27	Laßnitzhöhe	0.18	St. Lorenzen am Wechsel	0.14
Deutschlandsberg	0.1	Lebring-St. Margarethen	0.25	St. Lorenzen im Mürztal	0.23
Dobl-Zwaring	0.14	Leibnitz	0.19	St. Marein-Feistritz	0.14
Ebersdorf	0.32	Leoben	0.12	St. Marein bei Graz	0.21
Edelsbach bei Feldbach	0.24	Leutschach an der Weinstraße	0.25	St. Marein im Mürztal	0.26
Edelschrott	0.15	Lieboch	0.13	St. Margarethen an der Raab	0.2
Eggersdorf bei Graz	0.25	Liezen	0.34	St. Margarethen bei Knittelfeld	0.13
Ehrenhausen an der Weinstraße	0.16	Ligist	0.15	St. Martin am Wöllmißberg	0.14
Eibiswald	0.17	Lobmingtal	0.14	St. Martin im Sulmtal	0.16
Eichkögl	0.18	Ludersdorf-Wilfersdorf	0.17	St. Michael in Obersteiermark	0.12
Eisenerz	<0.1	Maria Lankowitz	0.14	St. Nikolai im Sausal	0.2
Empersdorf	0.21	Mariazell	0.18	St. Oswald bei Plankenwarth	0.15
Fehring	0.26	Markt Hartmannsdorf	0.19	St. Peter-Freienstein	0.12
Feistritztal	0.36	Mautern in der Steiermark	<0.1	St. Peter am Kammersberg	0.18
Feldbach	0.25	Mettersdorf am Saßbach	0.25	St. Peter am Ottersbach	0.23
Feldkirchen bei Graz	0.14	Miachaelerberg-Pruggern	0.12	St. Peter im Sulmtal	0.15
Fernitz-Mellach	0.16	Miesenbach bei Birkfeld	0.17	St. Peter ob Judenburg	0.2
Fischbach	0.16	Mitterberg-St. Martin	0.16	St. Radegund bei Graz	<0.1
Fladnitz an der Teichalm	0.15	Mittersdorf an der Raab	0.12	St. Ruprecht an der Raab	0.12
Floing	0.15	Mooskirchen	0.16	St. Stefan im Rosental	0.22
Fohnsdorf	0.18	Mortantsch	0.17	St. Stefan ob Leoben	0.14
Frauental an der Laßnitz	0.1	Mühlen	0.32	St. Stefan ob Stainz	0.15
Friedberg	0.14	Murau	0.18	St. Veit in der Südsteiermark	0.23
Frohnleiten	0.23	Mureck	0.25	Stadl-Predlitz	0.2
Fürstenfeld	0.2	Mürzzuschlag	0.13	Stainach-Pürgg	0.44
Gaal	0.18	Naas	0.12	Stainz	0.16
Gabersdorf	0.2	Nestelbach bei Graz	0.19	Stallhofen	0.16
Gaishorn am See	0.17	Neuburg an der Mürz	0.12	Stanz im Mürztal	0.27
Gamlitz	0.21	Neudau	0.22	Stattegg	0.1
Gasen	0.17	Neumarkt in der Steiermark	0.28	Stiwoll	0.15
Geistthal-Södingberg	0.27	Niederwölz	0.23	Straden	0.26
Gersdorf an der Feistritz	0.2	Niklasdorf	0.11	Strallegg	0.14
Gleinstätten	0.22	Obdach	0.15	Straß in Steiermark	0.17
Gleisdorf	0.15	Oberhaag	0.24	Stubenberg	0.16
Gnas	0.51	Oberwölz	0.22	Teufenbach-Katsch	0.23
Gössendorf	0.17	Öblarn	0.24	Thal	0.17
Grafendorf bei Hartberg	0.32	Ottendorf an der Rittschein	0.14	Thannhausen	0.11
Gralla	0.18	Paldau	0.23	Thörl	0.12
Gratkorn	0.14	Passail	0.14	Tieschen	0.21
Gratwein-Straßengel	0.14	Peggau	0.24	Tillmitsch	0.18
Graz	0.15	Pernegg an der Mur	0.17	Traboch	0.1
Greinbach	0.29	Pinggau	0.17	Tragöß-St. Katharein	0.14
Gröbming	0.14	Pirching am Traubenberg	0.25	Trieben	0.17
Groß St. Florian	0.12	Pischelsdorf am Kulm	0.31	Trofaiach	<0.1
Großklein	0.18	Pölfing-Brunn	0.14	Turnau	0.12
Großsteinbach	0.36	Pöllau	0.16	Übelbach	0.45
Großwilfersdorf	0.19	Pöllauberg	0.15	Unterlamm	0.26
Grundlsee	0.11	Pöls-Oberkurzheim	0.16	Unzmarkt-Frauenburg	0.22
Gutenberg-Stenzengreith	0.13	Pölstal	0.17	Vasoldsberg	0.21
Halbenrain	0.22	Preding	0.21	Voitsberg	0.55
Hart bei Graz	0.2	Premstätten	0.15	Vorau	0.15
Hartberg	0.34	Proleb	0.12	Vordernberg	<0.1
Hartberg Umgebung	0.35	Puch bei Weiz	0.15	Wagna	0.21
Hartl	0.3	Pusterwald	0.18	Wald am Schoberpaß	<0.1
Haseldorf-Tobelbad	0.19	Raaba-Grambach	0.27	Waldbach-Mönichwald	<0.1
Haus	0.3	Radmer	<0.1	Weinitzen	0.1
Hausmannstätten	0.15	Ragnitz	0.23	Weißkirchen in der Steiermark	0.13
Heiligenkreuz am Waasen	0.27	Ramsau am Dachstein	0.3	Weiz	0.14
Heimschuh	0.22	Ranten	0.17	Wenigzell	0.14
Hengsberg	0.22	Ratten	0.12	Werndorf	0.17
Hirschegg-Pack	0.28	Rettenegg	0.14	Wettmannstätten	0.26
Hitzendorf	0.21	Riegersburg	0.23	Wies	0.14
Hofstätten an der Raab	0.17	Rohr bei Hartberg	0.33	Wildalpen	0.16
Hohentauern	0.17	Rohrbach an der Lafnitz	0.18	Wildon	0.23
Ilz	0.25	Rosental an der Kainach	0.14	Wörschach	0.4
Ilztal	0.24	Rottenmann	0.16	Wundschuh	0.19
Irdning-Donnersbachtal	0.47	Schäffern	0.17	Zeltweg	0.13
Jagerberg	0.23	Scheifling	0.23		
Judenburg	0.16	Schladming	0.25		
**Tyrol**					
Abfalterbach	0.23	Jenbach	0.11	Rohrberg	<0.1
Absam	0.12	Jerzens	0.42	Roppen	0.21
Achenkirch	<0.1	Jochberg	0.12	Rum	<0.1
Ainet	0.2	Jungholz	<0.1	Sautens	0.51
Aldrans	0.12	Kaisers	<0.1	Scharnitz	<0.1
Alpbach	<0.1	Kals am Großglockner	0.18	Schattwald	0.13
Amlach	<0.1	Kaltenbach	<0.1	Scheffau am Wilden Kaiser	<0.1
Ampass	0.21	Kappl	0.11	Schlaiten	0.22
Angath	<0.1	Karres	0.26	Schlitters	<0.1
Angerberg	<0.1	Karrösten	1.92	Schmirn	<0.1
Anras	0.17	Kartitsch	<0.1	Schönberg im Stubaital	0.25
Arzl im Pitztal	0.27	Kaunerberg	0.93	Schönwies	0.22
Aschau	<0.1	Kaunertal	0.13	Schwaz	<0.1
Assling	0.13	Kauns	0.86	Schwendau	<0.1
Aurach bei Kitzbühl	<0.1	Kematen in Tirol	0.11	Schwendt	<0.1
Außervillgraten	0.13	Kirchberg in Tirol	<0.1	Schwoich	<0.1
Axams	0.25	Kirchbichl	<0.1	See	0.34
Bach	<0.1	Kirchdorf in Tirol	0.24	Seefeld in Tirol	0.15
Bad Häring	<0.1	Kitzbühel	<0.1	Sellrain	0.14
Baumkirchen	0.15	Kolsass	<0.1	Serfaus	<0.1
Berwang	0.2	Kolsassberg	0.17	Sillian	<0.1
Biberwier	1.26	Kössen	0.18	Silz	0.85
Bichlbach	0.18	Kramsach	<0.1	Sistrans	0.1
Birgitz	0.13	Kufstein	<0.1	Sölden	0.12
Brandberg	<0.1	Kundl	0.1	Söll	<0.1
Brandenberg	<0.1	Ladis	0.18	Spiss	<0.1
Breitenbach am Inn	0.1	Landeck	0.13	St. Anton am Arlberg	0.14
Breitenwang	0.12	Längenfeld	0.2	St. Jakob in Defereggen	0.24
Brixen im Thale	0.13	Langkampfen	<0.1	St. Jakob in Haus	<0.1
Brixlegg	<0.1	Lans	0.13	St. Johann im Walde	0.14
Bruck am Ziller	<0.1	Lavant	<0.1	St. Johann in Tirol	<0.1
Buch in Tirol	0.1	Lechaschau	0.19	St. Leonhard im Pietztal	0.21
Dölsach	0.23	Leisach	0.19	St. Sigmund im Stubaital	0.24
Ebbs	<0.1	Lermoos	0.2	St. Ulrich am Pillersee	0.15
Eben am Achensee	<0.1	Leutasch	0.19	St. Veit in Defereggen	0.14
Ehenbichl	0.15	Lienz	0.16	Stams	0.31
Ehrwald	<0.1	Mariastein	<0.1	Stans	0.23
Elbigenalp	0.1	Matrei am Brenner	<0.1	Stanz bei Landeck	0.16
Ellbögen	<0.1	Matrei in Osttirol	0.1	Stanzach	<0.1
Ellmau	<0.1	Mayrhofen	<0.1	Steeg	<0.1
Elmen	0.99	Mieders	<0.1	Steinach am Brenner	<0.1
Erl	0.17	Mieming	<0.1	Steinberg am Rofan	<0.1
Faggen	0.34	Mils	<0.1	Strass im Zillertal	<0.1
Fendels	0.11	Mils bei Imst	0.15	Strassen	0.17
Fieberbrunn	0.1	Mötz	0.18	Strengen	0.15
Finkenberg	0.1	Münster	<0.1	Stumm	0.11
Fiss	0.14	Musau	0.11	Stummerberg	<0.1
Flaurling	0.28	Mutters	<0.1	Tannheim	0.2
Fließ	0.1	Namlos	0.19	Tarrenz	0.16
Flirsch	0.18	Nassereith	0.31	Telfes im Stubai	<0.1
Forchach	<0.1	Natters	0.22	Telfs	0.27
Fritzens	0.24	Nauders	<0.1	Terfens	<0.1
Fügen	<0.1	Navis	<0.1	Thaur	0.18
Fügenberg	0.1	Nesselwängle	0.15	Thiersee	0.12
Fulpmes	<0.1	Neustift im Stubaital	0.15	Thurn	0.13
Gaimberg	0.1	Niederndorf	0.1	Tobadill	<0.1
Gallzein	<0.1	Niederndorferberg	0.24	Tösens	0.5
Galtür	<0.1	Nikolsdorf	0.16	Trins	<0.1
Gerlos	<0.1	Nußdorf-Debant	0.16	Tristach	0.19
Gerlosberg	<0.1	Oberhofen im Inntal	0.61	Tulfes	<0.1
Gnaderwald	0.19	Oberlienz	0.16	Tux	<0.1
Going am Wilden Kaiser	<0.1	Obernberg am Brenner	<0.1	Uderns	<0.1
Götzens	<0.1	Oberndorf in Tirol	<0.1	Umhausen	0.37
Gramais	0.11	Oberperfuss	<0.1	Unterperfuss	0.28
Grän	<0.1	Obertilliach	0.14	Untertilliach	0.13
Gries am Sellrain	0.16	Obsteig	0.11	Vals	<0.1
Gries am Brenner	0.19	Oetz	0.12	Vils	0.24
Grins	0.18	Patsch	0.1	Virgen	<0.1
Grinzens	0.1	Pettnau	0.24	Volders	0.12
Gschnitz	0.13	Pettneu am Arlberg	0.21	Völs	0.11
Haiming	<0.1	Pfaffenhofen	0.34	Vomp	<0.1
Hainzenberg	<0.1	Pfafflar	0.19	Vorderhornbach	0.19
Hall in Tirol	0.14	Pflach	0.1	Waidring	0.22
Hart im Zillertal	0.14	Pfunds	0.72	Walchsee	0.17
Häselgehr	0.11	Pians	0.11	Wängle	0.15
Hatting	0.59	Pill	<0.1	Wattenberg	0.1
Heinfels	<0.1	Pinswang	0.15	Wattens	<0.1
Heiterwang	<0.1	Polling in Tirol	0.41	Weer	<0.1
Hinterhornbach	<0.1	Prägraten am Großvenediger	<0.1	Weerberg	<0.1
Hippach	<0.1	Prutz	0.36	Weißenbach am Lech	0.14
Hochfilzen	<0.1	Radfeld	<0.1	Wenns	0.13
Höfen	0.18	Ramsau im Zillertal	<0.1	Westendorf	<0.1
Holzgau	0.11	Ranggen	0.62	Wiesing	<0.1
Hopfgarten im Brixental	<0.1	Rattenberg	0.12	Wildermieming	0.11
Hopfgarten in Defereggen	0.1	Rattenschöss	0.12	Wildschönau	<0.1
Imst	0.16	Reith bei Kitzbühel	<0.1	Wörgl	<0.1
Imsterberg	0.24	Reith bei Seefeld	0.11	Zams	0.2
Innervillgraten	0.49	Reith im Alpbachtal	<0.1	Zell am Ziller	0.13
Innsbruck	0.14	Reutte	0.21	Zellberg	0.14
Inzing	1.09	Ried im Oberinntal	0.11	Zirl	0.42
Ischgl	0.11	Ried im Zillertal	0.1	Zöblen	0.11
Iselsberg-Stronach	0.12	Rietz	1		
Itter	<0.1	Rinn	0.1		
**Upper Austria**					
Adlwang	<0.1	Kirchham	<0.1	Rosenau am Hengstspaß	0.25
Aichkirchen	0.2	Kirchheim im Innkreis	0.19	Roßbach	0.14
Aigen-Schlägl	0.11	Kirchschlag bei Linz	<0.1	Roßleithen	<0.1
Aistersheim	0.27	Klaffer am Hochficht	0.13	Rottenbach	0.16
Alberndorf in der Riedmark	0.13	Klam	0.34	Rüstorf	<0.1
Alkoven	0.16	Klaus an der Pyhrnbahn	<0.1	Rutzenham	<0.1
Allerheiligen im Mühlkreis	0.32	Kleinzell im Mühlkreis	0.12	Sandl	0.1
Allhaming	0.11	Kollerschlag	0.11	Sarleinsbach	<0.1
Altenberg bei Linz	0.15	Königswiesen	0.19	Sattledt	<0.1
Altenfelden	0.17	Kopfing im Innkreis	<0.1	Saxen	0.23
Altheim	0.1	Kremsmünster	<0.1	Schalchen	<0.1
Altmünster	0.1	Krenglbach	0.1	Schardenberg	0.19
Altschwendt	0.19	Kronstorf	0.13	Schärding	0.2
Ampflwang im Hausruckwald	0.14	Laakirchen	<0.1	Scharnstein	<0.1
Andorf	0.17	Lambach	0.1	Scharten	0.18
Andrichsfurt	0.16	Lambrechten	0.17	Schenkenfelden	<0.1
Ansfelden	0.22	Langenstein	0.45	Schiedlberg	0.13
Antiesenhofen	0.2	Lasberg	0.18	Schildorn	0.15
Arbing	0.3	Laussa	0.12	Schlatt	0.26
Arnreit	0.15	Lembach im Mühlkreis	<0.1	Schleißheim	<0.1
Aschach an der Donau	0.16	Lengau	0.17	Schlierbach	<0.1
Aschach an der Steyr	0.2	Lenzing	<0.1	Schlüßlberg	0.28
Aspach	<0.1	Leonding	0.18	Schönau im Mühlkreis	0.59
Asten	0.15	Leopoldschlag	0.17	Schörfling am Attersee	<0.1
Attersee am Attersee	0.14	Lichtenau im Mühlkreis	0.1	Schwand im Innkreis	0.16
Attnang-Pucheim	0.1	Lichtenberg	0.1	Schwanenstadt	<0.1
Atzbach	<0.1	Liebenau	0.13	Schwarzenberg am Böhmerwald	<0.1
Atzesberg	0.11	Linz	0.16	Schwertberg	0.55
Auberg	0.11	Lochen am See	<0.1	Seewalchen am Attersee	0.1
Auerbach	0.1	Lohnsburg am Kobernaußerwald	0.13	Senftenbach	0.15
Aurach am Hongar	<0.1	Losenstein	0.11	Sierning	0.13
Aurolzmünster	0.15	Luftenberg an der Donau	0.39	Sigharting	0.14
Bachmanning	0.31	Manning	<0.1	Sipbachzell	<0.1
Bad Goisern am Hallstättersee	<0.1	Marchtrenk	0.14	Sonnberg im Mühlkreis	<0.1
Bad Hall	0.13	Maria Neustift	0.1	Spital am Phyrn	<0.1
Bad Ischl	<0.1	Maria Schmolln	<0.1	St. Aegidi	0.17
Bad Kreuzen	0.13	Mattighofen	0.11	St. Agatha	0.17
Bad Leonfelden	<0.1	Mauerkirchen	<0.1	St. Florian	0.17
Bad Schallerbach	0.23	Mauthausen	0.29	St. Florian am Inn	0.16
Bad Wimsbach-Neydharting	<0.1	Mayrhof	0.13	St. Georgen am Fillmannsbach	<0.1
Bad Zell	0.14	Meggenhofen	0.15	St. Georgen am Walde	0.21
Baumgartenberg	0.24	Mehrnbach	0.16	St. Georgen an der Gusen	0.39
Berg im Attergau	<0.1	Mettmach	0.14	St. Georgen bei Grieskirchen	0.26
Braunau am Inn	0.16	Michaelnbach	0.24	St. Georgen bei Obernberg am Inn	0.17
Brunnenthal	0.18	Micheldorf in Oberösterreich	0.14	St. Georgen im Attergau	<0.1
Buchkirchen	0.15	Mining	0.1	St. Gotthard im Mühlkreis	0.16
Burgkirchen	<0.1	Mitterkirchen im Machland	0.22	St. Johann am Walde	0.15
Desselbrunn	0.78	Molln	0.13	St. Johann am Wimberg	<0.1
Diersbach	0.28	Mondsee	0.11	St. Konrad	<0.1
Dietach	0.14	Moosbach	0.12	St. Leonhard bei Freistadt	0.61
Dimbach	0.22	Moosdorf	<0.1	St. Lorenz	0.19
Dorf an der Pram	0.18	Mörschwang	0.21	St. Marien	0.15
Ebensee am Traunsee	0.1	Mühlheim am Inn	0.16	St. Marienkirchen am Hausruck	0.14
Eberschwang	0.1	Munderfing	0.1	St. Marienkirchen an der Polsenz	0.23
Eberstalzell	0.13	Münzbach	0.39	St. Marienkirchen bei Schärding	0.15
Edlbach	0.24	Münzkirchen	0.12	St. Martin im Innkreis	0.11
Edt bei Lambach	0.1	Naarn im Machlande	0.38	St. Martin im Mühlkreis	0.15
Eferding	0.17	Natternbach	0.12	St. Nikola an der Donau	<0.1
Eggelsberg	0.1	Nebelberg	<0.1	St. Oswald bei Freistadt	0.1
Eggendorf im Traunkreis	0.13	Neufelden	0.14	St. Oswald bei Haslach	0.12
Eggerding	0.27	Neuhofen an der Krems	0.2	St. Pankraz	<0.1
Eidenberg	<0.1	Neuhofen im Innkreis	0.18	St. Pantaleon	0.1
Eitzing	0.13	Neukirchen am Walde	0.11	St. Peter am Hart	0.12
Engelhartzell	<0.1	Neukirchen an der Enknach	0.11	St. Peter am Wimberg	0.12
Engerwitzdorf	0.37	Neukirchen an der Vöckla	0.13	St. Radegund	0.13
Enns	0.21	Neukirchen bei Lambach	0.13	St. Roman	0.14
Enzenkirchen	<0.1	Neumarkt im Hausruckkreis	0.22	St. Stefan-Afiesl	<0.1
Eschenau im Hausruckkreis	0.12	Neumarkt im Mühlkreis	0.45	St. Thomas	0.24
Esternberg	<0.1	Neustift im Mühlkreis	0.1	St. Thomas am Blasenstein	0.22
Feldkirchen an der Donau	0.14	Niederkappel	0.12	St. Ulrich bei Steyr	0.14
Feldkirchen bei Mattighofen	0.11	Niederneukirchen	0.14	St. Ulrich im Mühlkreis	0.12
Fischlham	<0.1	Niederthalheim	0.13	St. Veit im Innkreis	0.14
Fornach	<0.1	Niederwaldkirchen	0.14	St. Veit im Mühlkreis	0.14
Fraham	0.18	Nußbach	<0.1	St. Willibald	0.15
Frankenburg am Hausruck	<0.1	Nußdorf am Attersee	0.18	St. Wolfgang im Salzkammergut	<0.1
Frankenmarkt	<0.1	Oberhofen am Irrsee	0.12	Stadl-Paura	0.16
Franking	<0.1	Oberkappel	0.11	Steegen	0.19
Freinberg	0.11	Obernberg am Inn	0.24	Steinbach am Attersee	0.15
Freistadt	<0.1	Oberndorf bei Schwanenstadt	0.1	Steinbach am Ziehberg	0.11
Gaflenz	0.17	Oberneukirchen	<0.1	Steinbach an der Steyr	0.1
Gallneukirchen	0.26	Oberschlierbach	0.2	Steinerkirchen an der Traun	<0.1
Gallspach	0.21	Obertraun	<0.1	Steinhaus	0.36
Gampern	0.1	Oberwang	0.16	Steyr	0.16
Garsten	0.14	Oepping	0.15	Steyregg	0.2
Gaspoltshofen	0.17	Offenhausen	0.2	Straß im Attergau	0.13
Geboltskirchen	0.13	Oftering	0.15	Stroheim	0.16
Geiersberg	0.15	Ohlsdorf	<0.1	Suben	0.16
Geinberg	0.21	Ort im Innkreis	0.21	Taiskirchen im Innkreis	0.16
Geretsberg	<0.1	Ostermiething	0.14	Tarsdorf	0.1
Gilgenberg am Weilhart	0.13	Ottenschlag im Mühlkreis	0.12	Taufkirchen an der Pram	0.18
Gmunden	0.13	Ottensheim	0.17	Taufkirchen an der Trattnach	0.11
Goldwörth	0.14	Ottnang am Hausruck	0.1	Ternberg	0.16
Gosau	0.13	Pabneukirchen	0.14	Thalheim bei Wels	0.15
Gramastetten	<0.1	Palting	<0.1	Tiefgraben	0.12
Grein	0.25	Pasching	0.18	Timelkam	0.1
Grieskirchen	0.24	Pattingham	0.17	Tollet	0.25
Großraming	0.18	Peilstein im Mühlviertel	0.13	Tragwein	0.19
Grünau im Almtal	0.13	Pennewang	0.15	Traun	0.17
Grünbach	<0.1	Perg	0.28	Traunkirchen	0.17
Grünburg	<0.1	Perwang am Grabensee	0.12	Treubach	0.21
Gschwandt	<0.1	Peterskirchen	0.12	Tumeltsham	0.15
Gunskirchen	0.2	Pettenbach	0.11	Überackern	0.21
Gurten	0.14	Peuerbach	0.24	Ulrichsberg	0.13
Gutau	0.37	Pfaffing	<0.1	Ungenach	0.14
Haag am Hausruck	<0.1	Pfaffstätt	0.11	Unterach am Attersee	0.18
Hagenberg im Mühlkreis	0.46	Pfarrkirchen bei Bad Hall	0.1	Unterweißenbach	0.37
Haibach im Mühlkreis	<0.1	Pfarrkirchen im Mühlkreis	<0.1	Unterweitersdorf	0.46
Haibach ob der Donau	0.11	Piberbach	0.13	Utzenaich	0.19
Haigermoos	0.13	Pichl bei Wels	0.33	Vichtenstein	<0.1
Hallstatt	<0.1	Pierbach	0.26	Vöcklabruck	0.1
Handenberg	<0.1	Pilsbach	0.17	Vöcklamarkt	<0.1
Hargelsberg	0.11	Pinsdorf	<0.1	Vorchdorf	<0.1
Hartkirchen	0.21	Pischelsdorf am Engelbach	0.12	Vorderstoder	<0.1
Haslach an der Mühl	0.12	Pitzenberg	0.11	Vorderweißenbach	<0.1
Heiligenberg	0.23	Pollham	0.24	Waizenkirchen	0.21
Helfenberg	<0.1	Polling im Innkreis	0.12	Waldburg	0.46
Hellmonsödt	0.12	Pöndorf	0.17	Waldhausen im Strudengau	0.73
Helpfau-Uttendorf	0.12	Pötting	0.19	Walding	0.16
Herzogsdorf	0.13	Pram	0.38	Waldkirchen am Wesen	<0.1
Hinterstoder	<0.1	Prambachkirchen	0.16	Waldneukirchen	0.11
Hinzenbach	0.21	Pramet	0.12	Waldzell	0.12
Hirschbach im Mühlkreis	0.38	Pregarten	0.49	Wallern an der Trattnach	0.12
Hochburg-Ach	0.13	Puchenau	0.2	Wartberg an der Krems	<0.1
Hofkirchen an der Trattnach	0.24	Puchenkirchen am Trattberg	0.1	Wartberg ob der Aist	0.51
Hofkirchen im Mühlkreis	0.23	Pucking	0.1	Weibern	0.14
Hofkirchen im Traunkreis	0.15	Pühret	0.11	Weilbach	0.16
Hohenzell	0.14	Pupping	0.1	Weißenkirchen an der Traun	0.13
Höhnhart	<0.1	Putzleinsdorf	0.1	Weißenkirchen im Attergau	<0.1
Holzhausen	0.17	Raab	0.18	Weitersfelden	0.12
Hörbich	<0.1	Rainbach im Innkreis	0.47	Wels	0.1
Hörsching	0.16	Rechberg	0.45	Wendling	0.38
Innerschwand am Mondsee	0.2	Redleiten	0.15	Weng im Innkreis	0.11
Inzersdorf im Kremstal	<0.1	Redlham	0.15	Wernstein am Inn	0.21
Jeging	0.1	Regau	<0.1	Weyer	0.14
Julbach	<0.1	Reichenau im Mühlkreis	<0.1	Weyregg am Attersee	0.11
Kallham	0.2	Reichenthal	0.12	Wilhering	0.34
Kaltenberg	0.23	Reichersberg	0.63	Windhaag bei Freistadt	0.1
Katsdorf	0.41	Reichraming	0.18	Windhaag bei Perg	0.42
Kefermarkt	0.26	Reinbach im Mühlkreis	0.2	Windischgarsten	0.57
Kematen am Innbach	<0.1	Ried im Innkreis	0.16	Wippenham	0.16
Kematen an der Krems	0.17	Ried im Traunkreis	<0.1	Wolfern	0.1
Kirchberg bei Mattighofen	0.14	Ried in der Riedmark	0.5	Wolfsegg am Hausruck	<0.1
Kirchberg ob der Donau	0.11	Riedau	0.16	Zell am Moos	0.14
Kirchberg-Thening	0.18	Rohr im Kremstal	0.16	Zell am Pettenfirst	0.15
Kirchdorf am Inn	0.16	Rohrbach-Berg	0.12	Zell an der Pram	0.18
Kirchdorf an der Krems	0.17	Roitham am Traunfall	<0.1	Zwettl an der Rodl	<0.1
**Carinthia**					
Afritz am See	0.18	Gurk	0.18	Poggersdorf	0.19
Albeck	0.16	Guttaring	0.24	Pörtschach am Wörthersee	0.19
Althofen	0.19	Heiligenblut am Großglockner	0.24	Preitenegg	0.15
Arnoldstein	0.18	Hermagor-Pressegger See	0.40	Radenthein	0.21
Arriach	0.16	Himmelberg	0.18	Rangersdorf	0.25
Bad Bleiberg	0.23	Hohenthurn	0.15	Reichenau	0.17
Bad Kleinkirchheim	0.22	Hüttenberg	0.26	Reichenfels	0.26
Bad St. Leonhard im Lavanttal	0.10	Irschen	0.30	Reißeck	0.12
Baldramsdorf	0.18	Kappel am Krappfeld	0.19	Rennweg am Katschberg	0.11
Berg im Drautal	0.20	Keutschach am See	<0.1	Rosegg	0.22
Bleiburg	<0.1	Kirchbach	0.16	Ruden	0.29
Brückl	0.27	Klagenfurt am Wörthersee	0.32	Sachsenburg	0.12
Dellach	0.10	Kleblach-Lind	0.13	Schiefling am Wörthersee	0.17
Dellach im Drautal	0.27	Klein St. Paul	0.13	Seeboden am Millstätter See	0.20
Deutsch-Griffen	0.16	Kötschach-Mauthen	0.12	Sittersdorf	<0.1
Diex	0.25	Köttmannsdorf	<0.1	Spittal an der Drau	0.20
Ebenthal in Kärnten	0.10	Krems in Kärnten	0.13	St. Andrä	0.15
Eberndorf	0.16	Krumpendorf am Wörthersee	0.21	St. Georgen am Längsee	0.31
Eberstein	0.20	Lavamünd	0.19	St. Georgen im Lavanttal	0.17
Eisenkappel-Vellach	0.16	Lendorf	0.11	St. Jakob im Rosental	0.10
Feistritz an der Gall	0.20	Lesachtal	0.11	St. Kanzian am Klopeiner See	0.23
Feistritz im Rosental	<0.1	Liebenfels	0.20	St. Paul im Lavanttal	0.20
Feistritz ob Bleiburg	<0.1	Ludmannsdorf	0.11	St. Stefan im Gailtal	0.18
Feld am See	0.18	Lurnfeld	0.96	St. Urban	0.15
Feldkirchen in Kärnten	0.21	Magdalensberg	0.34	St. Veit an der Glan	0.22
Ferlach	<0.1	Mallnitz	0.20	Stall	0.23
Ferndorf	0.22	Malta	0.15	Steindorf am Ossiacher See	0.21
Finkenstein am Faaker See	0.20	Margareten im Rosental	<0.1	Steinfeld	0.11
Flattach	0.22	Maria Rain	<0.1	Steuerberg	0.14
Frantschach-St. Gertraud	0.13	Maria Saal	0.34	Stockenboi	0.27
Frauenstein	0.32	Maria Wörth	<0.1	Straßburg	0.19
Fresach	0.24	Metnitz	0.23	Techelsberg am Wörther See	0.17
Friesach	0.17	Micheldorf	0.35	Trebesing	0.24
Gallizien	0.16	Millstatt am See	0.19	Treffen am Ossiacher See	0.14
Gitschtal	0.41	Mölbling	0.20	Velden am Wörther See	0.21
Glanegg	0.20	Moosburg	0.20	Villach	0.17
Globasnitz	0.23	Mörtschach	0.26	Völkermarkt	0.10
Glödnitz	0.17	Mühldorf	0.11	Weißensee	0.39
Gmünd in Kärnten	0.13	Neuhaus	0.22	Weißenstein	0.19
Gnesau	0.15	Nötsch im Gailtal	0.20	Weitensfeld im Gurktal	0.15
Grafenstein	0.21	Oberdrauburg	0.30	Wernberg	0.24
Greifenburg	0.10	Obervellach	0.25	Winklern	0.23
Griffen	0.26	Ossiach	0.25	Wolfsberg	0.11
Großkirchheim	0.26	Paternion	0.23	Zell	<0.1
**Salzburg**					
Abtenau	<0.1	Hollersbach	<0.1	Salzburg	<0.1
Adnet	<0.1	Hüttau	<0.1	Scheffau	0.17
Altenmarkt	<0.1	Hüttschlag	<0.1	Schleedorf	<0.1
Anif	<0.1	Kaprun	<0.1	Schwarzach	<0.1
Annaberg	0.17	Kleinarl	<0.1	Seeham	<0.1
Anthering	<0.1	Koppl	<0.1	Seekirchen	<0.1
Bad Gastein	0.17	Köstendorf	<0.1	St. Andrä	0.36
Bad Hofgastein	<0.1	Krimml	<0.1	St. Georgen	<0.1
Bergheim	<0.1	Krispl	<0.1	St. Gilgen	<0.1
Berndorf	<0.1	Kuchl	<0.1	St. Johann	<0.1
Bischofshofen	<0.1	Lamprechtshausen	<0.1	St. Koloman	<0.1
Bramberg	0.16	Lend	<0.1	St. Margarethen	<0.1
Bruck	<0.1	Leogang	<0.1	St. Martin	<0.1
Bürmoos	<0.1	Lessach	<0.1	St. Martin bei Lofer	<0.1
Dienten	<0.1	Lofer	<0.1	St. Michael	<0.1
Dorfbeuern	<0.1	Maishofen	<0.1	St. Veith	<0.1
Dorfgastein	<0.1	Maria Alm	<0.1	Straßwalchen	<0.1
Eben	<0.1	Mariapfarr	<0.1	Strobl	<0.1
Ebenau	<0.1	Mattsee	<0.1	Stuhlfelden	<0.1
Elixhausen	<0.1	Mauterndorf	<0.1	Tamsweg	<0.1
Elsbethen	<0.1	Mittersill	<0.1	Taxenbach	<0.1
Eugendorf	<0.1	Mühlbach	<0.1	Thalgau	<0.1
Faistenau	<0.1	Muhr	<0.1	Thomatal	<0.1
Filzmoos	<0.1	Neukirchen	0.2	Tweng	<0.1
Flachgau	<0.1	Neumarkt	<0.1	Unken	<0.1
Forstau	<0.1	Niedernsill	0.12	Unternberg	0.12
Fusch	<0.1	Nußdorf	<0.1	Untertauern	0.516
Fuschl am See	<0.1	Oberalm	<0.1	Uttendorf	<0.1
Goldegg	<0.1	Oberndorf	<0.1	Viehhofen	<0.1
Golling	<0.1	Obertrum	<0.1	Vigaun	<0.1
Göming	<0.1	Pfarrwerfen	<0.1	Wagrain	<0.1
Göriach	<0.1	Piesendorf	<0.1	Wald im Pinzgau	<0.1
Grödig	<0.1	Plainfeld	<0.1	Wals-Siezenheim	<0.1
Großarl	<0.1	Puch	<0.1	Weißbach b. Lofer	<0.1
Großgmain	<0.1	Radstadt	<0.1	Weißpriach	<0.1
Hallein	<0.1	Ramingstein	<0.1	Werfen	<0.1
Hallwang	<0.1	Rauris	<0.1	Werfenweng	<0.1
Henndorf	<0.1	Rußbach	<0.1	Zederhaus	<0.1
Hintersee	<0.1	Saalbach-Hinterglemm	<0.1	Zell am See	<0.1
Hof b. Salzburg	<0.1	Saalfelden	<0.1		
**Burgenland**					
Andau	<0.1	Kittsee	<0.1	Podersdorf	<0.1
Apetlon	<0.1	Kohfidisch	<0.1	Purbach a. Neusiedlersee	<0.1
Bad Sauerbrunn	<0.1	Königsdorf	<0.1	Rechnitz	<0.1
Bad Tatzmannsdorf	<0.1	Kukmirn	<0.1	Rust	<0.1
Bernstein	<0.1	Lockenhaus	<0.1	Schachendorf	<0.1
Breitenbrunn	<0.1	Lutzmannsburg	<0.1	Schützen	<0.1
Burg	<0.1	Mannersdorf a.d. Rabnitz	<0.1	Sieggraben	<0.1
Deutschkreuz	<0.1	Markt St. Martin	<0.1	St.Andräe am Zicksee	<0.1
Donnerskirchen	<0.1	Mattersburg	<0.1	St.Magarethen im Burgenland	<0.1
Eberau	<0.1	Mönchhof	<0.1	St.Michael im Burgenland	<0.1
Eisenstadt	<0.1	Moschendorf	<0.1	Stegersbach	<0.1
Frauenkirchen	<0.1	Neufeld a. d. Leitha	<0.1	Stoob	<0.1
Gols	<0.1	Neusiedl am See	<0.1	Strem	<0.1
Großpetersdorf	<0.1	Nikitsch	<0.1	Tadten	<0.1
Güssing	<0.1	Oberpullendorf	<0.1	Trausdorf a.d. Wulka	<0.1
Heilgenkreuz i. Lafnitztal	<0.1	Oberschützen	<0.1	Wallern	<0.1
Hornstein	<0.1	Oberwart	<0.1	Weiden am See	<0.1
Ilmitz	<0.1	Oggau	<0.1	Weppersdorf	<0.1
Jennersdorf	<0.1	Pamhagen	<0.1	Zurndorf	<0.1
Jois	<0.1	Parndorf	<0.1		
Kemeten	<0.1	Pinkafeld	<0.1		
**Vorarlberg**					
Alberschwende	<0.1	Götzis	<0.1	Reuthe	<0.1
Altach	<0.1	Hard	<0.1	Riefensberg	<0.1
Andelsbuch	<0.1	Hittisau	<0.1	Röns	<0.1
Au	<0.1	Höchst	<0.1	Röthis	<0.1
Bartholomäberg	<0.1	Hohenems	<0.1	Satteins	<0.1
Bezau	<0.1	Hohenweiler	<0.1	Schlins	0.20
Bildstein	<0.1	Hörbranz	<0.1	Schnepfau	<0.1
Bizau	<0.1	Innerbraz	0.33	Schnifis	<0.1
Blons	<0.1	Kennelbach	<0.1	Schoppernau	<0.1
Bludenz	0.22	Klaus	<0.1	Schröcken	<0.1
Bludesch	<0.1	Klösterle	0.21	Schruns	0.54
Brand	<0.1	Koblach	<0.1	Schwarzach	<0.1
Bregenz	<0.1	Krumbach	<0.1	Schwarzenberg	<0.1
Buch	<0.1	Langen bei Bregenz	<0.1	Sibratsgfäll	<0.1
Bürs	<0.1	Langenegg	<0.1	Silbertal	0.38
Bürserberg	<0.1	Laterns	<0.1	Sonntag	<0.1
Dalaas	<0.1	Lauterach	<0.1	St. Anton im Montafon	0.35
Damüls	<0.1	Lech	<0.1	St. Gallenkirch	0.51
Doren	<0.1	Lingenau	<0.1	St. Gerold	<0.1
Dornbirn	<0.1	Lochau	<0.1	Stallehr	0.27
Düns	<0.1	Lorüns	0.14	Sulz	0.14
Dünserberg	<0.1	Ludesch	<0.1	Sulzberg	<0.1
Egg	<0.1	Lustenau	<0.1	Thüringen	<0.1
Eichenberg	<0.1	Mäder	<0.1	Thüringerberg	<0.1
Feldkirch	0.13	Meiningen	0.12	Tschagguns	0.15
Fontanella	<0.1	Mellau	<0.1	Übersaxen	<0.1
Frastanz	<0.1	Mittelberg	<0.1	Vandans	0.15
Fraxern	0.11	Möggers	<0.1	Viktorsberg	<0.1
Fußach	<0.1	Nenzing	0.12	Warth	<0.1
Gaißau	<0.1	Nüziders	0.16	Weiler	<0.1
Gaschurn	<0.1	Raggal	<0.1	Wolfurt	<0.1
Göfis	0.16	Rankweil	<0.1	Zwischenwasser	<0.1
**Vienna**					
Alserbachstraße	<0.1				
Anton Böck Gasse	<0.1				
Große Stadtgutgasse	<0.1				
